# Hospital preferences of nursing students in Korea: a discrete choice experiment approach

**DOI:** 10.1186/s12960-016-0156-1

**Published:** 2016-09-29

**Authors:** Bo-hyun Park, YuKyung Ko

**Affiliations:** 1 Department of Nursing, Changwon National University, Changwon, 51140 Republic of Korea; 2 Department of Nursing, College of Medicine, Wonkwang University, 460 Iksandae-ro, Iksan, Jeonbuk 54538 Republic of Korea

**Keywords:** Career choice, Nursing students, Rural health services, Salaries and fringe benefits, Discrete choice experiment

## Abstract

**Background:**

DCE was applied to investigate nursing students’ preferred hospital choice criteria and to investigate the trends in the trade-offs by calculating the marginal rate of substitution between these criteria. This study identified the properties of the hospitals primarily selected by nursing students, and aims to estimate the monetary value of each attribute.

**Methods:**

Based on discussions and in-depth interviews with nursing students’ focus groups and a literature review, we created a discrete choice experiment (DCE) that assessed how students’ stated preference for a certain hospital choice was influenced by various job attributes: higher salary, location, hospital type, salary per year, provision of a dormitory, etc. We applied this DCE to nursing students in South Korea using a brief structured questionnaire, and we used conditional logit models to estimate the utility of each job’s attributes. Willingness to pay (WTP) was estimated as the ratio of the value of the coefficient of interest to the negative of the cost attribute.

**Results:**

Complete data for the DCE analysis were available for 702 nursing students. In the main effect mixed logit model, the welfare system and organizational culture were most strongly associated with job preference. Location, hospital type, and opportunity to upgrade qualifications had a negative influence on hospital choice. The WTP threshold was 7,043,000 KRW for the welfare system and 9,928,000 KRW for the organizational culture (relation-oriented).

**Conclusions:**

Better nursing working conditions, such as a positive organizational culture and the provision of a welfare system, can improve the motivation and applications for hospitals in rural areas.

## Background

The decrease in the number of health care providers is the most important problem in the health care system today. Nursing shortages are exacerbated by factors that appear as regional differences. In most countries, in rural or underserved areas, recruiting and then retaining nurses and health care personnel is not an easy task [1]. One of the biggest demands of patients living in rural areas or far away from hospitals is to receive high-quality medical care from experienced and competent health care providers in a fair way [2]. In particular, it is very important to have competent nurses to take care of patients day and night, for 24 h, in various situations that threaten the safety and health of people, including new infectious diseases and unpredicted disasters. However, this is seldom realized in practice. A uniform distribution of competent health care providers is important, and when this is not met, inequality in health outcomes is likely to arise [3]. Accordingly, having competent nurses is very important not only in Seoul and surrounding areas but also in rural areas, and policies that can attract health care providers, including nurses and doctors, to hospitals located in rural or remote areas are in high demand. Previous studies on various attributes that attract health care providers to hospitals in rural areas have been conducted on a wide scale; these studies have suggested the creation of a more supportive work environment, provision of financial incentives, and improvements in the educational opportunities for employees [4–6].

Instead of market competition based on the quality of medical service, bipolarization has been prominent in the medical market in Korea: most patients visit primary hospitals, which have higher accessibility and competitiveness in terms of price, and tertiary hospitals, which provide high-quality medical services [7]. In particular, the proportion of patients from rural areas has been increasing in the Four Big Hospitals in Seoul, which are the Seoul Asan Medical Center, Yonsei University Severance Hospital, Seoul Samsung Medical Center, and Seoul National University Hospital. According to the recent report on Issues and Improving Strategies on Korea Healthcare Delivery System conducted by the Korea Institute for Health and Social Affairs, the 2013 medical expense of the Five Big Hospitals of Korea, 2290.3 billion KRW, accounted for 7.8 % of the medical expense of all hospitals and for 35.7 % of the medical expense of all tertiary hospitals [8]. The bipolarization of the use of medical services leads to bipolarization in the distribution of health care providers, translating in quantitative and qualitative inequality in nursing human resources and also as inequality in the regional distribution of nurses. In other words, with increases in the number of large-scale general hospitals, competition between hospitals to secure nurses has become intense and the number of nurses that transfer from hospitals in rural areas to hospitals in Seoul and the surrounding areas in search of a higher salary and welfare benefits has increased. According to the 2013 survey conducted on 201 hospitals in Korea by the Hospital Nurses Association, the turnover rate of Korean nurses, assessed based on the data from late December 2013, was 13.9 %, on average. The turnover rate was higher in smaller hospitals, and a high turnover rate, 28.8 %, was observed in hospitals with less than 200 beds. In contrast, the lowest turnover rate, 10.0 %, was observed in hospitals with more than 1000 beds [9].

Although many nursing students, soon to become nurses, still cannot find their desired job, it can be said that we have entered an era wherein hospitals with satisfying nursing environments, meeting the expectations of nurses, are primarily selected. Moreover, when medical consumers are not satisfied with the services provided by a hospital, they avoid that hospital, thereby curbing the pay ability of that hospital [7]. In medical markets with low price elasticity of demand, as in Korea, hospitals compete with each other in terms of the quality and quantity of the medical service in order to attract more patients [10]. Therefore, hospitals are striving to hire competent nurses by satisfying nurses’ needs through the provision of nursing environments that are distinguished from other hospitals. Since nursing students—that is, future nurses—do not have accurate information on work environments provided to nurses by hospitals, including various work and welfare benefits, students tend to choose hospitals based on location, rather than on the core attributes that influence their work environment. In other words, the career decision of nurses is made depending on additional attributes, including whether the hospital is located in large cities, small cities, or rural areas. However, studies that discussed in detail the criteria used by nursing students in choosing hospitals are almost absent. Therefore, the criteria used by future nurses in choosing hospitals should be investigated.

In this regard, an evaluation of the effectiveness of various policies is required. However, evaluating interventions in human resource management (HRM) is quite different from drug tests intended for the evaluation of the drugs’ effectiveness [11]. In other words, although government officials formulating policies expect HRM policies to be conducted under more regulated environments, it is difficult to apply such concepts in reality. Moreover, the evidence on the statistical significance achieved in well-controlled experiments may be insufficient to provide information on operational policy decision-making [12]. Of course, financial incentives provided by rural hospitals do indeed contribute to employment and retention of nurses in such areas. However, due to the various circumstances prevalent at rural hospitals, finances are often constrained. Furthermore, policy makers require detailed information with regard to practical demands: they require information on the cost-effectiveness and relative impact of human resource intervention under various circumstances, wherein multiple choice criteria exist [12].

When making any complex decision, to make a reasonable choice, multiple criteria should be considered at the same time. The discrete choice experiment (DCE) is a way to reflect various choice criteria simultaneously [13]. DCE randomly combines more than two attributes and attribute levels to build hypothetical scenarios and has the respondents choose their preferred scenarios to determine the relative importance of the attributes [14]. DCE has recently begun receiving attention as a tool to evaluate the relative impact of attributes chosen by consumers or to identify the criteria used by health care providers in choosing hospitals, in particular the criteria used in allocating scarce health care resources, and thus, it is widely used in the research on health and medical services [12, 15–20]. In Korea, DCE has been used in business administration to analyze the hospital preference of medical consumers [7] and it has also been utilized to analyze the criteria used to determine health insurance benefits on medications and medical supplies from the perspective of insurers [16]. However, DCE has not been used yet to analyze the distribution of hospital nurses in South Korea. According to studies conducted in other countries, which used DCE to analyze the hospital preference of health care providers, including nurses, salary (a monetary attribute) was found to exert an absolute influence on hospital choice. However, non-monetary attributes, such as the provision of dormitories, well-maintained facilities and infrastructure, opportunities for education and training, and early promotion, were also shown to be important attributes [12, 15–20].

Although the monetary value of non-monetary attributes was also suggested in the aforementioned studies, due to currency differences between Korea and the countries in which the studies were conducted, it is difficult to directly compare the results. The willingness to pay (WTP), the marginal rate of substitution of non-monetary attributes for attributes with monetary value, was high for the following attributes: in Kosltad’s study, WTP was the highest for hospitals that provided educational opportunities, followed by hospitals with well-maintained facilities and infrastructure [18], while in Rockers et al.’s study, WTP was the highest in hospitals with well-maintained facilities, followed by hospitals that provided support on educational expenses and those with supportive administrators [20]. An analysis using DCE is, thus, attempted in this study to investigate the relative effectiveness of the various methods used by rural hospitals to hire nurses. By investigating the preference of nursing students in the decision-making criteria used to choose hospitals, this study aims to provide the necessary information required to rationalize and clarify the career decision-making criteria for nursing students and to provide basic data that can be used by rural hospitals in developing strategies to attract nurses. Moreover, by suggesting various policies that can be applied to rural hospitals based on the attributes considered by nursing students in their hospital choice, this study will suggest various strategies and alternative measures to solve the issue of concentration of nurses in certain hospitals.

### Purpose of the study

The purposes of the present study are as follows. This study identified the properties of the hospitals primarily selected by nursing students and aims to estimate the monetary value of each attribute. To this end, first, we investigate the attributes and attribute levels related to hospital choice among nursing students. Second, using a DCE, we investigate the attributes that influence the hospital choice of nursing students. Third, we investigate the trade-off trends among hospital choice criteria.

## Methods

### Discrete choice experiment

When multiple choice options on alternatives are available, people compare the utility of each alternative and choose the one with the highest utility. The consumer theory assumes that individuals make choices to maximize their own reasonable and stable preference systems, and discrete choice experiment (DCE) is an analysis method based on this assumption [21]. DCE can be applied to macroeconomic aspects of health care systems, such as the distribution of resources to maximize social convenience, but it can be also applied to microeconomic issues to explain the hospital choice of patients or health care providers. In this study, we aim to conduct DCE to elucidate the major attributes considered in the hospital choice process of nursing students. Nursing students decide the hospitals they will work at by comparing the characteristics of each hospital. Therefore, to conduct a DCE, various attributes related to the hospital choice of nursing students are first defined, and then, the levels of each attribute are compared. After determining the attributes and attribute levels, multiple hypothetical alternatives are suggested and respondents’ choices are observed. The alternatives suggested here are shown as combinations of attributes that are shortlisted after a detailed investigation, and the attributes in each alternative are set to have different levels. The influence of the attributes that determine the value of the alternatives is identified through an attribute-dependent alternative choice model, based on information on both the chosen and rejected alternatives.

### Determination of the attributes and attribute levels used in the DCE scenario

We conducted a literature review of the attributes used in previous studies, which were carried out in other countries to analyze nurses’ hospital preferences using DCE. Mangham and Hanson [19] used location, salary, dormitory, facilities and infrastructure, educational training, and promotion opportunities as attributes. In the study of Blaauw et al. [12], hospital type, salary, educational training, dormitory, promotion, additional salary, and organizational culture were used as attributes. In the study of Hanson and Jack [22], location, salary, dormitory, facilities and infrastructure, educational training, permission on private-sector work, and supervision of work were used as attributes. Kruk et al. [17] used salary, education for children, medical facilities and infrastructure, management style, years of work before study leave, provision of a dormitory, and provision of transportation as attributes. Based on the findings of previous studies, we initially set two levels over six attributes: geographical location, salary per year, usefulness of material resources, workload, provision of a dormitory, and opportunity to upgrade qualifications. Attributes should be identified as the main factors considered by nursing students in making their career decisions. Therefore, we confirmed the significance of attributes and attribute levels through two sets of interviews that were conducted on six and eight current nursing students, respectively. Two nursing professors conducted a final review on the attributes and attribute levels deduced through the literature review and student interviews; usefulness of material resources and workload were excluded from the analysis, and seven attributes, including the additionally selected hospital type, welfare system, and organizational culture, were selected.

Attribute levels were determined upon consideration of the literature review findings and the levels currently being applied in hospitals. The location of hospitals was classified into “metropolitan areas and large cities,” “medium or small cities,” and “rural or residence area,” and hospital type was classified into “tertiary hospitals” and “general hospitals.” Salary per year was divided into brackets of “25,000,000 KRW,” “35,000,000 KRW,” and “45,000,000 KRW.” Provision of a dormitory was divided into “yes” and “no,” and opportunity to upgrade qualifications was classified into “between 3 and 5 years” and “more than 5 years.” Welfare system, which refers to parental leave, assistance on educational expenses, and provision of time and financial assistance for leisure activities, was classified into “sufficient” and “insufficient.” Organizational culture was divided into “hierarchy-oriented” organizational culture and “relation-oriented” organizational culture (Table 1).

**Table 1:** Attributes used in the DCE as influencing nursing students’ stated job preference

Attributes	Levels	Coding	Dummy variable
Location	Capital area or large city	1	0, 1
	Medium or small city	2	1, 0
	Rural or residence area	3	0, 0
Hospital type	Tertiary hospitals	1	1
	General hospitals	2	0
Salary per year (unit: 1,000,000 KRW)	25,000,000 KRW	2.5	2.5
	35,000,000 KRW	3.5	3.5
	45,000,000 KRW	4.5	4.5
Provision of dormitory	Yes	1	1
	No	2	0
Opportunity to upgrade qualifications	3~5 years	1	1
	More than 5 years	2	0
Welfare system	Sufficient	1	1
	Insufficient	2	0
Organizational culture	Hierarchy-oriented	1	0
	Relations-oriented	2	1

### Questionnaire preparation

Through the DCE design, we identified scenarios that combined different attributes. Since there were 5 attributes with two levels and 2 attributes with three levels, 2^5^ * 3^2^ = 288 full factorial designs are required. However, it is impossible to suggest all these alternatives for the respondents to choose. By extracting only a few of these alternatives, optimal choice sets, which can make effective estimations practically equivalent to full factorial designs, can be created. Huber and Zwerina [23] suggested the following requirements of optimal choice sets: (1) level balance, meaning that levels of each attribute appear at equal frequencies in choice sets; (2) orthogonality, meaning that levels of each attribute change independently; (3) repetition of an attribute level in alternatives within each choice set should be minimized; and (4) alternatives in each choice set should be equally attractive. Accordingly, we extracted 15 choice sets with high statistical utility through the %mktruns, %mktex, and %choiceff functions of the SAS statistical package. After excluding 2 choice sets that were judged to be unrealistic, 13 choice sets were selected. Then, we added general characteristics to the final 13 choice sets to complete the questionnaire (Table 2). When correlations between each pair of variables in the final questionnaire were analyzed, the correlation coefficient (*r*) did not exceed 0.3, thereby satisfying the requirement of independence. Moreover, since the frequency of each attribute level was similar, the aforementioned requirements of the choice experiment were satisfied.

**Table 2:** Sample of DCE card and script presented to nursing students

Choice set 1	Hospital A	Hospital B
Location of the hospital	Metropolitan area	Local, residence area
Hospital type	Tertiary hospital	General hospital
Annual salary	35,000,000 KRW	25,000,000 KRW
Provision of dormitory	Yes	No
Opportunity to upgrade qualifications	More than 5 years	3~5 years
Benefit plans	Insufficient	Sufficient
Organizational culture	Relation-oriented	Relation-oriented
In your opinion, of the two hospitals described, which one do you think is the best hospital?	Hospital A	Hospital B

### Subjects of study and data collection

This study was conducted on students currently enrolled in nursing programs. According to previous studies, subjects of studies that used DCE to investigate the hospital preference of health care providers included various jobs, such as doctors, nurses, midwives, pharmacists, and medical laboratory scientists. Among these, doctors and nurses were the subjects of many studies; studies have been conducted not only on currently practicing doctors and nurses but also on medical school students and nursing students [24]. Since this study is the first attempt of its kind in Korea, nursing students were selected as subjects of study. Upon consideration of regional differences, data were collected after dividing the subjects into students enrolled in universities located in Seoul and the surrounding areas and those studying in universities located in other areas. We expected that the accessibility and preference for large hospitals would differ between students living in Seoul and the surrounding areas and those living in other areas, since large hospitals in Korea are mostly concentrated in Seoul and the surrounding areas. Therefore, we accounted for this aspect when recruiting the subjects of the study.

Data were collected from 710 students from four schools—two in Seoul and the surrounding areas and two in other areas—over a period of 8 weeks, between March 2 and April 30, 2015. A total of 702 students responded, with a response rate of 98.9 %. Before submitting the surveys, we explained the purpose of the study and provided information on the attributes included in the questionnaire. Respondents received explanations on each attribute and read and responded to the questionnaire autonomously. Among the respondents, 25.8 % (181) were studying at universities located in Seoul and the surrounding areas and 74.2 % (521) were enrolled in universities in other areas.

### Analysis model and methods

The random utility model is the theoretical basis for DCE data analysis [25]. In this model, the utility of a given job, which is a dependent variable, is a combination of two components: a deterministic component comprising the function of observable job attributes and a random component (i.e., an error term) comprising the function- and individual-level variations in unobserved job attributes. The regression coefficients obtained from the model can be used for two purposes. First, coefficients can determine the importance of attributes through statistical significance, the direction of their effect, and the relative importance through the size of the estimated parameters. Second, through the direction of the coefficient, the theoretical or internal validity of the DCE model can be confirmed. In other words, the regression coefficients show whether the results agree with previous research findings. Moreover, the value calculated through DCE is used to investigate two aspects: first, when continuous variables are included, a quantitative estimation on the trade-off between attributes is feasible. If the continuous variable is salary, the ratio of the coefficient of a given attribute to price proxy can be calculated, and this ratio indicates the WTP of various job attributes. Second, the probability of taking up defined posts can be estimated. Since this indicates the predicted influence of newly offered alternative jobs on levels of job attributes, this finding is very useful for policy makers. For instance, Hanson and Jack [22] reported that providing double salaries in non-metropolitan areas could increase the supply of nurses from 4 to 27 %.

Previous studies applied the probit model, which assumes a normal distribution of the error term [19, 22, 25], or applied the logit model, which assumes a logistic distribution [18]. Due to its flexible applicability, the logit model is preferred in studies that use DCE. In this study, the subjects choose one hospital of their preference among given choice sets consisting of hospitals A and B. We used the conditional logit model as an analytic tool, a type of multinomial model that can be used when a choice is made among multiple alternatives. The conditional logit model analyzes the relationship between the characteristics of alternatives (when different values for each of multiple alternatives are present) and response variables. The predicted probability of choosing one among multiple alternatives can be explained as below:

$$ {P}_i=\frac{ \exp \left({V}_i\right)}{{\displaystyle \sum_{j=1}^j \exp \left({V}_j\right)}} $$

Generally, the conditional logit analysis assumes independence of irrelevant alternatives (IIA). This assumption implies that the odds ratio between the choice probabilities of the two choice alternatives stays consistent, regardless of the presence of other choice alternatives. In other words, this means that, when a new alternative is included in the choice set, the choice probability of this alternative can take away a certain portion of the choice probability of the existing alternatives. In this study, since we assumed that the utility related to hospital A was the same as the utility related to hospital B, conditional logit functions can be used.

The willingness to pay (WTP), which refers to the maximum amount a person is willing to pay for a certain attribute, can be measured directly or indirectly. The DCE is an indirect measurement method of the WTP, calculated through the marginal rate of substitution, which is the ratio of the coefficient of each attribute to the coefficient of the salary attribute. This indicates respondents’ WTP for improving certain attributes among hospital choice attributes. WTP reflects the portion of monthly salary that respondents are willing to sacrifice for an aspect of an attribute rather than for other aspects. Within the context of workforce issues, the inclusion of a price proxy (such as salary) allows the researcher to estimate the monetary value of the attributes of a job, that is, how much salary a respondent would be willing to give up to achieve an improvement over other aspects of the job [24].

$$ \mathrm{W}\mathrm{T}\mathrm{P}\left({\mathrm{location}}_{\mathrm{Seoul}\ \mathrm{and}\ \mathrm{surrounding}\ \mathrm{areas},\ \mathrm{large}\ \mathrm{cities}}\right)= - \frac{\vartheta U/\vartheta \left({\mathrm{location}}_{\mathrm{Seoul}\ \mathrm{and}\ \mathrm{surrounding}\ \mathrm{areas},\ \mathrm{large}\ \mathrm{cities}}\right)}{\vartheta U/\vartheta \mathrm{salary}}=-\frac{\beta_2}{\beta_1} $$

### Ethical considerations

This study was conducted upon approval of the University Institutional Review Board (review number: IRB-201406-SB-037). Prior to data collection, researchers visited each school seeking participation through phone calls. Then, the purpose and methods of the study were explained to the deans of the schools, and their cooperation was sought. After explaining the rights of the subjects of the study, confidentiality clauses, and purposes of the study to the chosen subjects, questionnaires were distributed. The subjects responded to questions in the structured questionnaire, which took approximately 15 min.

## Results

Whit regard to the general characteristics of the investigated subjects, there were more female respondents (625; 89.0 %) than male respondents (77; 11.0 %). In terms of academic year, third-year students were the majority (237; 33.8 %), followed by fourth-year students (207; 29.5 %), first-year students (131; 18.7 %), and second-year students (126; 17.9 %). Most students reported their perceived socioeconomic status as “middle” (600; 85.5 %). With regard to the geographical location, more students lived in district towns (495, 70.5 %) than in metropolitan areas (207; 29.5 %) (Table 3).

**Table 3:** Demographic characteristics of nursing students in the DCE (*n* = 702)

Characteristics	Number	Percent
Gender		
Male	77	11.0
Female	625	89.0
Grade	699	
1st	131	18.7
2nd	126	17.9
3rd	237	33.8
4th	207	29.5
Missing data	1	0.1
Social economic status	699	
Low	79	11.3
Medium	600	85.5
High	22	3.1
Missing data	1	0.1
Location		
District town	495	70.5
Metropolitan area	207	29.5

When differences in attribute levels were analyzed, statistically significant coefficients were obtained for the following attributes: salary, geographical location, hospital type, opportunity to upgrade qualifications, welfare system, and organizational culture. In other words, except for the provision of a dormitory, statistical significance was observed in all other attributes (Table 4). The statistical significance of the coefficient on attributes means that the level of each attribute influences the subjects’ choice of scenarios; in other words, the subjects considered salary, geographical location, hospital type, opportunity to upgrade qualifications, welfare system, and organizational culture as important when choosing hospitals. In particular, the hospital choice probability was higher when the salary was higher, and the choice probability was lower in metropolitan or large-, medium-, or small-sized cities compared to areas near the students’ areas of residence. The probability of choosing tertiary hospitals was lower than the probability of choosing general hospitals, and the probability of choosing hospitals that require 3–5 years to upgrade qualifications was lower than the probability of choosing hospitals that require more than 5 years to upgrade qualifications. The probability of choosing hospitals that provide a sufficient welfare system was higher, and the probability of choosing hospitals with relation-oriented organizational culture was higher than the probability of choosing hospitals with hierarchy-oriented organizational culture (*P* < .001).

**Table 4:** Results of logit model and WTP for job attributes

Attributes	Coefficients	SE	*P*	95 % CI		WTP	95 % CI	
Salary, net yearly pay (unit: 1 000 000 KRW)	0.134	2.48E−03	<.001	0.129	0.139			
Urban scale (ref: rural or residence area)								
Metropolitan area	−0.722	0.045	<.001	−0.810	−0.634	−5.385	6.028	4.741
Small city	−0.587	0.044	<.001	−0.673	−0.501	−4.378	5.025	3.730
Hospital type (ref: general hospital)								
Tertiary hospital	−0.122	0.037	.001	−0.194	−0.050	−0.911	1.445	0.377
Provision of dormitory (ref: none)								
Yes	0.023	0.036	.520	−0.047	0.093	0.172	0.353	0.697
Opportunity to upgrade qualifications (ref: more than 5 years)								
3–5 years	−0.298	0.038	<.001	−0.373	−0.223	−2.222	2.773	1.670
Benefit plan (ref: insufficiency)								
Sufficient	0.944	0.038	<.001	0.869	1.020	7.043	6.472	7.614
Organizational culture (ref: hierarchical-oriented)								
Relations-oriented	1.331	0.039	<.001	1.255	1.407	9.928	9.372	10.484
Number of respondents	702							
Number of observations	18 045							
Log likelihood	5375.86							
Pseudo *R* ^2^	.24							

Dependent variable = 1, if choice of job A or job B = 1

*CI* confidence interval, *SE* standard error, *WTP* willingness to pay

**Fig. 1:**
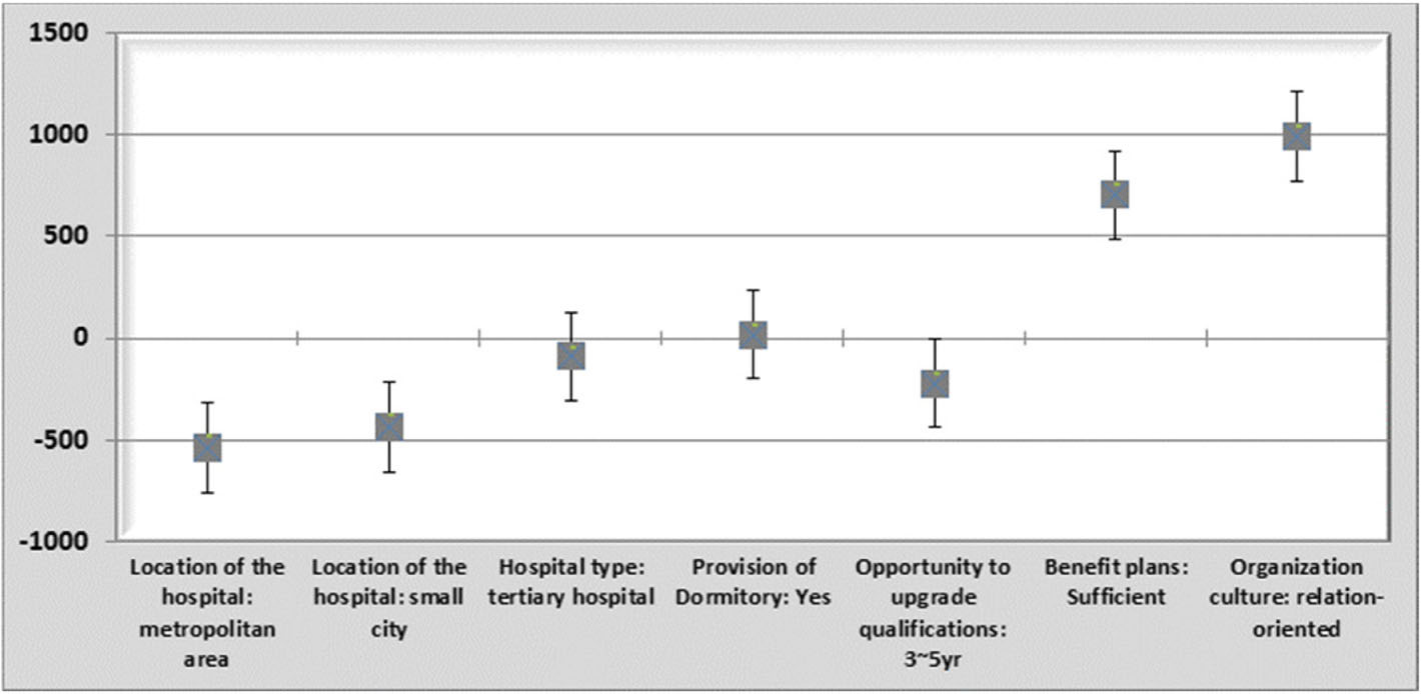
WTP estimates and 95 % confidence intervals for job attributes for nursing students

Using the ratio of the coefficients of each attribute calculated from the model, the trends of the trade-off between attribute levels were analyzed. Through the ratios of the coefficients of each attribute, the marginal rate of substitution or WTP was calculated, which indicates the amount that respondents are willing to pay to improve the level of certain attributes. According to the findings of this study, nursing students were willing to pay 7,043,000 KRW to choose hospitals that provide a sufficient welfare system. Moreover, students were willing to pay 9,928,000 KRW to choose hospitals with relation-oriented organizational culture. Furthermore, the subjects would be willing to choose hospitals in large cities, as opposed to those in rural areas or their areas of residence, if they were to receive 5,385,000 KRW. In addition, they would be willing to choose tertiary hospitals, as opposed to general hospitals, only if they were to receive 911,000 KRW. Similarly, the subjects would choose hospitals that require 3–5 years to upgrade qualifications, as opposed to those that require more than 5 years, if they were to receive 2,222,000 KRW. Figure 1 provides a graphical representation of the WTP values.

## Discussion

In this study, DCE was applied to investigate nursing students’ preferred hospital choice criteria and to investigate the trends in the trade-offs among criteria by calculating their marginal rate of substitution. To conduct a DCE, as a first step, various attributes and attribute levels related to the hospital choice of nursing students should be defined. After determining the attributes and attribute levels, multiple hypothetical alternatives are suggested, and respondents’ choices are observed. Therefore, the determination of the attributes and levels is a very important part of this study. The selected attributes and attribute levels had been modified through two rounds of focus group interviews. The attributes and attribute levels used in this study were considered to be reliable because their selecting process was carried out in compliance with the DCE procedure.

We found that nursing students preferred hospitals that provided a sufficient welfare system and had a relation-oriented organizational culture. The criterion regarding the provision of housing, such as a dormitory, was the only attribute that was not statistically significant. Although statistical significance was observed in the following attributes, the direction of the coefficient was the opposite of what we expected: statistical significance was observed in hospitals in rural areas and areas of students’ residence rather than those in large-, medium-, and small-sized cities, in general hospitals rather than tertiary hospitals, and in hospitals that require more than 5 years to upgrade qualifications rather than those that require 3–5 years. This may be attributed to most subjects (70.5 %, 495) living in rural areas, and given their personal circumstances, students in rural areas are likely to prefer their current areas of residence or nearby areas. Moreover, the fact that opportunities to work at tertiary hospitals, including famous tertiary hospitals, such as the Five Big Hospitals, are provided only to a small number of students with good grades, this is likely to have prevented the majority of students from choosing tertiary hospitals from the beginning. Initially, when we conducted two sets of interviews with nursing students to extract the attributes related to their hospital choice, we observed that students in rural areas were worried about the difficulties of hospital work, living alone in other areas, and adapting to intense competition, which would be potential concerns when they find jobs at large hospitals in big cities. Therefore, we can deduce that students in rural areas will choose large hospitals in big cities only when a certain amount of compensation for such psychological burden is provided. Both internal and external rewards, such as new adaptation programs, recreation fee, and vacation pay support to alleviate these psychological burdens, are necessary. However, the interviews were conducted to extract the relevant factors: if the location of a school is far away geographically, and this factor is reported in two different schools, this can be seen as a limitation of the present study.

Kruk et al. [17] used DCE to analyze whether fourth-year medical school students in Ghana preferred rural postings accompanied by various attributes including high salary, free superior housing, provision of educational expenses for children, improved facilities and infrastructure, and supportive management. As a result, improved facilities and infrastructure and supportive management had high correlations with job preference, and the interruption of provision of basic housing was shown to exert a negative influence. Moreover, better work environment was suggested to be related to the fourth-year Ghanaian medical school students’ choice of rural posting. Similar to the findings of Kruk et al., nursing students in this study were found to prefer hospitals that provide a sufficient welfare system and have a relation-oriented, rather than hierarchy-oriented, organizational culture [17], again confirming the importance of a favorable work environment.

In the study of Ko et al. [26], researchers investigated the attributes that influence innovative behaviors of general hospital nurses: age, organizational support, and hierarchical culture were found to exert influences on the nurses’ innovative behaviors. Since relation-oriented organizational cultures, rather than hierarchy-oriented organizational cultures, were shown to exert a stronger influence on hospital choice in the present study, our findings, thus, seem to be in contrast with those of Ko et al. [26]. Of course, since the subjects of Ko et al.’s study [26] were nurses, while our subjects were nursing students, a comparison should be made carefully. In particular, Ko et al. [26] pointed out that nursing organizations in Korea value hierarchical rankings due to the institutionalized organizational systems. We believe that nursing students may have been exposed to such an inflexible culture, which values hierarchical ranks, through clinical training or mass media and they may have been affected only by its negative aspects. Moreover, Ko et al. [26] also suggested that many nurses are choosing conventional behaviors and practices, rather than changes, since nurses that advocate for new opinions and changes are often perceived as challenging in the existing system. However, from the perspective of nursing students, such hierarchical culture, seen through clinical training, could have been perceived as inappropriate. Therefore, students could have preferred to choose hospitals with positive and appropriate relation-oriented organizational cultures, even if salaries are lower. The willingness of students to pay 9,930,000 KRW to choose hospitals with relation-oriented organizational cultures, rather than those with hierarchy-oriented organizational cultures, can also be interpreted in the same context.

In this study, the criterion related to the provision of housing, such as a dormitory, was the only attribute that was not statistically significant. Since Kruk et al.’s study [17] was conducted in Ghana, where hospitals are characterized by poor-quality housing, Ghanaian students could have suggested free basic housing as a requirement for rural posting; therefore, “no housing” was found to be very negatively correlated with hospital choice in Kruk et al.’s study [17]. In contrast, in Korea, even if nurses do not enter dormitories or the housing facilities provided by hospitals, they can easily find comfortable accommodations, such as studios and officetels (studio apartments in buildings with units for commercial and residential purposes), both in rural areas and in large cities. This is likely to have yielded the insignificant result on housing in this study. Similarly, our findings disagreed with other studies reporting that accommodation and housing were very important factors to encourage doctors or nurses in developing countries, such as Ghana and Ethiopia, to practice in rural areas [22, 27].

Preference for salary is related to the cost of living in other countries, and it is also related to the expected salary of graduates and their long-term career plans [17]. When Penn-Kekana et al. [28] analyzed South African nurses using a DCE, an increase in salary had the highest relative impact on hospital choice, although good management and well-equipped hospitals were also important attributes. When Mangham and Hanson [19] conducted a DCE to assess the employment of public nurses in Malawi, an increase in salary was suggested to improve not only the employment environment but also the motivation and retention rate of nurses. Since salary was found to be an attribute in hospital choice in this study as well, our findings were in line with those of previous studies. The reasons why Korean nurses avoid working in hospitals in rural areas are known to be the low salary and inadequate work environment of rural hospitals, especially medium- and small-sized hospitals. According to Lee and Cho [29], who analyzed changes in nurses’ salary, the differences in the salary of nurses employed in large-scale hospitals in Seoul and those that are employed in medium- and small-sized hospitals in other cities increased from 852,000 KRW in 2002 to 1,050,000 KRW in 2009. Furthermore, in the study of Park et al. [30], the annual salary of nurses working in Seoul and the surrounding areas was more than 3,000,000 KRW higher than that of nurses working in other metropolitan cities, which were already receiving 1,200,000 KRW more in comparison to nurses employed in other provincial cities. This study found that nursing students considered the organizational culture and welfare system when choosing hospitals, showing that non-monetary attributes are highly attractive to nursing students for choosing hospitals in rural areas. In this respect, the findings of the present study indicate important insights regarding the employment of Korean nurses. In other words, nursing administrators and hospital managers should take into account not only the salary but also non-monetary factors, such as a relationship-oriented organizational culture, in order to hire a qualified nurse. The advantage of DCE is that it can be used as a method to deduce the stated preference when useful information on real choice is absent [20]. Of course, this study has a few limitations. Since nursing students apply to hospitals, while hospitals choose to employ nursing students, and since the choice and employment used in the study differ from the reality, conducting DCE targeting nursing students has fundamental limitations. Therefore, we suggest conducting a preference analysis using DCE on turnover to other hospitals, targeting nurses currently employed in hospitals. Still, we believe that the present study provides useful information to policy makers. The results of this study will be able to suggest an effective policy to increase the employment level of the local hospital nurses. In addition, hospital executives may be introducing new policies towards the nursing staff to ensure the recognition of non-monetary factors in hospital choice. According to the results of the present study, all attributes except the provision of a dormitory were found to be significant. Interestingly, nursing students preferred hospitals that were close to their areas of residence, and they did not prefer tertiary hospitals and hospitals that offered early opportunities to upgrade qualifications. The attributes that were found to have the highest WTP were organizational culture and welfare system. In particular, in terms of organizational culture, students were found to be willing to pay approximately 10,000,000 KRW for relation-oriented organizational culture. Assuming that nurses at hospital A are paid 30,000,000 KRW per year, if this hospital has a relation-oriented organizational culture, this seems to have a similar effect as paying the nurses 40,000,000 KRW per year. Since this study confirmed that monetary values of non-monetary attributes are considerably high, a consideration of such attributes to improve the nursing work environment would be useful in a cost-effective management of human resources, in the future. However, since payment style (annual or monthly payments) can influence WTP, we suggest future studies to consider the effect of the payment style, period, and amount on such potential changes. Since the representativeness of the sample of the present study is limited, caution would be required in generalizing the findings of the present study to all nursing students in Korea. Based on the results of this study, further research is needed as follows. Non-monetary attributes should be addressed in more detail. Furthermore, it is necessary to conduct DCE using attributes and attribute levels identified in this study for nurses who already work in the field.

## Conclusions

This study analyzed the major attributes that influence the hospital choice of 702 nursing students with a DCE, and the study also calculated the WTP for improvements over each attribute, using the coefficients of the attributes obtained in the regression analysis. We found that salary, location of hospital, hospital type, opportunity to improve qualifications, welfare system, and organizational culture were the attributes that influence hospital choice. Nursing students were found to prefer hospitals with higher salary, relation-oriented organizational cultures, and a more-developed welfare system and hospitals that are close to their areas of residence. When the WTP for each attribute was analyzed, the relation-oriented organizational culture had the highest WTP, 9,928,000 KRW, followed by a sufficient welfare system, 7,043,000 KRW. Through this study, we could confirm that not only monetary attributes but also non-monetary attributes had a large influence on nursing students’ hospital preference. Moreover, the monetary values of non-monetary attributes were found to be considerably high. Therefore, in cases of hospitals with weak financial resources, the lack of nurses can be overcome in a cost-effective manner by improving non-monetary attributes with high monetary values. The findings of the present study would be useful for hospitals and medical service providers seeking to maintain and hire nurses under favorable circumstances, providing basic data for establishing marketing strategies on information provision for future nurses.

## Abbreviations

DCE: Discrete choice experiment
HRM: Human resource management
WTP: Willingness to pay
